# Detecting and monitoring lymphoma with high-throughput sequencing

**DOI:** 10.18632/oncotarget.270

**Published:** 2011-04-28

**Authors:** Harlan Robins

**Affiliations:** Fred Hutchinson Cancer Research Center, 1100 Fairview Ave N, Seattle, WA 98109

Each mature T cell or B cell has an allele with a productively rearranged T cell receptor (TCR) or B cell receptor (BCR) gene, respectively. The repertoire of TCRs and BCRs in the blood of a healthy person is diverse, with no single specific rearrangement found in more than one percent of cells, and most rearrangements representing a minute fraction of total B cells or T cells[1-3]. When a leukemia or lymphoma develops, one or a small number of specific clones proliferate rapidly. The cancerous clone (or clones) is “tagged” by the unique TCR or BCR sequences from the original cell. In leukemia patients, a single clone often accounts for over half of all B cells or T cells in the blood. The clonality of an adaptive immune receptors in the blood is often utilized in the diagnosis of lymphoma or leukemia. One of the two commonly utilized methods to track minimal residual disease (MRD) after treatment is to design a set of allele specific PCR primers that bind to the rearranged receptor in a specific cancerous clone[4].

High-throughput sequencing has the potential to significantly improve sensitivity and reduce cost for clonal detection and post-treatment monitoring of B and T cell leukemias and lymphomas. For leukemia, direct sequencing of BCR and TCR genes from blood cells is straightforward. However, in the case of lymphoma, the primary tumor resides in the lymph node, with the fraction of cancerous cells found in blood variable. A recent paper by He et al. presents the interesting idea that free DNA in blood plasma is potentially a better source of lymphoma specific DNA than white blood cells[5]. In the case of non-Hodgkins lymphoma (NHL), they show that the tumor specific BCR sequences can be detected in plasma. In addition, for an NHL cohort with plasma samples, but no corresponding tumor samples, they are able to identify a dominant rearranged BCR sequence in approximately half the patients, which is likely to be tumor derived DNA.

He et al. employ a targeted capture and sequencing approach that they call IgCap. It is similar to a shotgun sequencing approach, yet they include an enrichment step between fragmentation and sequencing which enriches for sequences derived from the IgH locus. Fragmented DNA sequences from plasma have adapters attached to each end, and are then hybridized to a set of probes that bind to the germline encoded fragments of the IgH locus. The subset of plasma DNA fragments which hybridize to the IgH derived probes are then sequenced using Illumina's high-throughput sequencing technology.

This methodology has some pros and cons. On the positive side, the method is capable of detecting the majority of sequences in the IgH locus, including any possible non-templated rearrangement. Importantly, IgCap was effectively able to detect and identify lymphoma specific IgH sequences in plasma. There are also a few negative aspects of this technology. First, the hybridization step does not distinguish between rearranged and non-rearranged IgH sequences. So, an unknown portion of the DNA fragments sequenced will truly be derived from the rearranged IgH loci. Second, fragmenting sequences and then reconstructing the pieces requires sequencing each receptor multiple times. For a diverse, non-templated region of DNA, such as the rearranged IgH locus, to detect an specific IgH sequence, many copies would need to be in the plasma and sequenced.

Direct methods for sequencing rearranged BCR and TCR genes have been developed over the past few years[2, 3, 6, 7]. These methods use primers specific to each pair of V and J segments and attempt to directly amplify and sequence all possible rearrangements. These methods avoid the cons of IgCap, but they have not been tested with plasma DNA. The IgCap method could be compared with the direct sequencing methods to determine which has greater sensitivity and also lower costs.

The work of He et al. is an important step in showing that high-throughput sequencing of the adaptive immune receptor repertoire has direct clinical application. The idea of focusing on plasma DNA instead of cells for monitoring lymphoma is intriguing. Hopefully, the larger scale experiments required to prove utility will be accomplished soon and these or similar methods will be moved into the clinic.
